# Evaluation of an alternative skeletal muscle index for skeletal muscle mass assessment in a group of Australian women

**DOI:** 10.1093/ageing/afac002

**Published:** 2022-02-12

**Authors:** Ming Li Yee, Sophie Einoder, Boyd J G Strauss, Christopher Gilfillan

**Affiliations:** Department of Endocrinology, Eastern Health, Victoria, Australia; Eastern Health Clinical School, Monash University, Victoria, Australia; Eastern Health Clinical School, Monash University, Victoria, Australia; School of Clinical Sciences, Faculty of Medicine, Nursing and Health Sciences, Monash University, Victoria, Australia; School of Medical Sciences, Division of Diabetes, Endocrinology and Gastroenterology, Faculty of Biology, Medicine and Health, The University of Manchester, Manchester, UK; Department of Endocrinology, Eastern Health, Victoria, Australia; Eastern Health Clinical School, Monash University, Victoria, Australia

**Keywords:** sarcopenia, skeletal muscle index, calf circumference, leg length, older people

## Abstract

**Background:**

Sarcopenia is assessed by several methods, including dual energy X-ray absorptiometry (DEXA), which provide a height-adjusted skeletal muscle index (H-SMI). A SMI 2 standard deviation below the young adult reference [1] combined with low muscle strength or performance is used to identify sarcopenia. As height declines with age, H-SMI may underestimate low skeletal muscle mass in the older population. Our study aims to evaluate an alternative SMI and to examine its relationship to grip strength in a group of Australian women.

**Methods:**

Women from two cohorts were analysed. 2041 women had body composition data (112 had calf circumference, 137 had leg length measurements) without grip strength, and 49 women had grip strength measured (40 had body composition data).The relationship between leg length-adjusted SMI (LL-SMI) to grip strength and anthropometric variables to skeletal muscle mass by DEXA were examined by linear regression analysis.

**Results:**

Cohort 1: Older women were compared to younger women. Older women were shorter but leg length did not differ between different age groups. H-SMI was not different between groups (*P* = 0.528). LL-SMI was lower in older women (*P* = 0.002). Cohort 2: LL-SMI was significantly associated with grip strength (*P* = 0.048) after adjustment for age.

**Conclusion:**

Older women were shorter, while leg length did not differ from the younger group. H-SMI may obscure and may underestimate low muscle mass in older individuals. LL-SMI may be a better measure of skeletal muscle mass in older individuals. These alternate SMI would benefit from further exploration in older individuals.

## Key Points

- Leg length was observed to remain unchanged with age and may serve as a useful denominator in the SMI.
- Calf circumference is a strong predictor of skeletal muscle mass.
- Height-adjusted skeletal muscle mass index may underestimate low skeletal muscle mass in the older population.

## Introduction

Sarcopenia is a loss in skeletal muscle mass and function with age. Following its initial description by Rosenberg [2], research has consistently demonstrated an association between sarcopenia with adverse outcomes, morbidity and mortality. While knowledge into this field has expanded in recent years, there remains a scarcity of studies in the Australian population.

In clinical practice, sarcopenia is identified by the presence of low skeletal muscle mass combined with low muscle performance or function. Skeletal muscle mass assessment is performed by a variety of imaging modalities, while low gait speed or grip strength are commonly used to assess muscle performance and function. The diagnosis of sarcopenia is dependent on the criteria proposed by different expert consensus groups [3–8].

Recommended gold standard imaging tools for skeletal muscle mass assessment are computed tomography scan and magnetic resonance imaging. Although these tools provide an accurate assessment of skeletal muscle mass, radiation dose and cost remain a limiting factor. As an alternative, the use of Dual energy X-ray absorptiometry (DEXA) is increasingly popular due to its low radiation exposure. Moreover, DEXA analysis provides information on a standardised skeletal muscle index (SMI), which is calculated by total appendicular lean mass (ALM) (kg) divided by standing height (m^2^). The use of the SMI allows further comparison between studies.

Increasing age is associated with a decline in height [9, 10]. This is due to a combination of loss of height of the vertebral column due to flattening of intervertebral tissues, loss of vertebral height due to crush fractures and increasing spinal curvature. Leg length remains unchanged. Given the use of height in the SMI, this may lead to underestimation of low muscle mass particularly in the older population where muscle loss is more prevalent. The lack of decline in height-adjusted SMI (H-SMI) with age has so far been reported in the Asian population [11, 12] particularly in older women [11]. To our knowledge, this has not been reported before in the Australian population.

Accurate height measurement in those with impaired mobility is another barrier to the use of H-SMI. Efforts to explore alternative measures for skeletal muscle mass assessment have led to the evaluation of anthropometric measures as another option. There is a good correlation between calf circumference (CC) with calf muscle mass [13–15], and reduced CC is associated with increased mortality [16–18]. In the Australian population, CC were predominantly associated with poor nutrition [19] and impaired function [20]; however, specific cut points to determine low skeletal muscle mass by CC measurement have not yet been identified.

The aims of our study were to evaluate an alternative SMI for skeletal muscle mass assessment and to examine its relationship to grip strength. We also compared anthropometry measures and examined their relationship to skeletal muscle mass by DEXA in a group of Australian women.

## Methods

Analysis was performed on women from two cohorts. A larger cohort (Cohort 1) of women was analysed for comparison of body composition and anthropometry and alternative SMI. As these women did not have measures of muscle strength, a smaller cohort (Cohort 2) with grip strength recorded was analysed to determine the relationship between the new leg length-adjusted SMI (LL-SMI) to grip strength. Description of both cohorts are provided:

### Cohort 1

Participants were recruited by convenience sampling between February 2001 and February 2012. This was a heterogeneous group of individuals who were predominantly referred for bone mineral density studies measured with Lunar DPX (General Electric, Madison, WI) or Lunar Prodigy (General Electric) dual energy absorptiometry (DEXA) machine. A smaller proportion of these women who were referred for more extensive body composition assessment had leg length and anthropometry recorded.

Estimated DEXA skeletal muscle mass (DEXA SMM) was calculated, using a regression equation model by Kim [21], on individuals with BMI between 15.9 and 35.0 kg/m^2^. To increase validity, only individuals who fall within this BMI range had DEXA SMM included in the analysis. Of the 2,434 women screened, 393 women with absent DEXA SMM or DEXA SMM < 8 kg were excluded, resulting in a total of 2,041 women analysed. Leg lengths calculated as a difference in sitting height and measured standing height were available from 137 women. Anthropometric measures were available from 112 women measured using methods outlined in Lohman [22]. This study was approved by the Monash University Human Research Ethics Committee Project ID 1158.

### Cohort 2

In this cohort, 49 participants were recruited between June 2016 and July 2019 for a separate study examining skeletal muscle mass in older women. This group consisted of 20 women with hip fracture, 10 women awaiting total hip replacement for osteoarthritis and 19 ambulant women from the community. This study was approved by the Eastern Health Ethics Committee HREC/16/EH/104.

Data from these women were analysed to report on the relationship between the conventional H-SMI and LL-SMI to muscle performance (grip strength). DEXA data were obtained by the Hologic Discovery Machine (New South Wales, Australia). ALMs in kg were calculated from the total lean mass in both arms and legs. In those who had hip surgery, DEXA assessment was performed in the post-operative period. Due to impaired mobility in women with acute hip fracture precluding accurate assessment of sitting height in this cohort, leg length was measured using the distance between the greater trochanter to the lateral malleolus either on the right leg or non-fracture leg. Grip strength assessment with a hand-held Jamar dynamometer was used as a measure of muscle performance, with an average of three attempts recorded as the final reading.

#### Height, weight and skeletal muscle mass index measurement

Height and weight were measured to the closest cm and kg in light clothing. SMI adjusted for height (H-SMI), leg length (LL-SMI) and fat mass index (FMI) were calculated, as follows:

H-SMI = ALM (kg)/height (m^2^),

LL-SMI = ALM (kg)/leg length (m^2^), and

FMI = total fat mass (kg)/height (m^2^).

Alternative skeletal muscle indices explored using leg lean mass and CC adjusted for height, leg length and knee height were calculated (Supplementary Table 1).

#### Low skeletal muscle mass by DEXA criteria

Reporting of low skeletal muscle mass by DEXA criteria were in reference to the European Working Group for Sarcopenia 2 [3] criteria, ALM/height^2^ (kg/m^2^) < 5.5 kg/m^2^.

### Statistical analysis

Analysis was performed with SPSS version 26 (IBM, Chicago). Parametric data were expressed as mean ± standard deviation and non-parametric data as median (interquartile range). Groupwise comparison, using Students *t*-test for parametric, Mann Whitney U test for non-parametric data and Chi square analysis for categorical variables, was performed. *P* value < 0.05 was selected to suggest the statistical significance. The relationships between anthropometric variables to DEXA SMM, and H-SMI or LL-SMI to grip strength were assessed in a linear regression analysis.

## Results

### Cohort 1

#### Demographics

Median age was 58 years old and range was 40–92 years. Women were divided into two age group categories for comparison: Group 1 (age: 40–60 years, *n* = 1,163) and Group 2 (age: 61–92 years, *n* = 878). Younger women were comparatively taller and had lower body mass index than older women (Supplementary Table 2). The majority of these women were referred for osteoporosis screening and follow-up for established osteoporosis (Supplementary Table 3).

##### Groupwise comparison (Cohort 1): Anthropometry

Older women were significantly shorter in standing and sitting height. Leg length was not significantly different between groups of younger and older women. Mid-upper arm, thigh and CC were significantly lower in older women (Supplementary Table 4).

##### Groupwise comparison (Cohort 1): Body composition

Older women when compared to the younger age group had low ALM, DEXA SMM and high FMI, and were more osteopenic (Supplementary Table 5). There was no difference in H-SMI between groups (*P* = 0.528). In the smaller group of women where leg length measurement was available (*n* = 137), LL-SMI was lower in older women (*P* = 0.002).

ALM adjusted for leg length and knee height, leg ALM adjusted for leg length and knee height and CC adjusted for knee height were significantly lower in older women. There was no significant difference in the proportion of individuals with low skeletal muscle mass by EWGSOP2 criteria (*P* = 0.971) (Supplementary Table 5).

**Figure 1:**
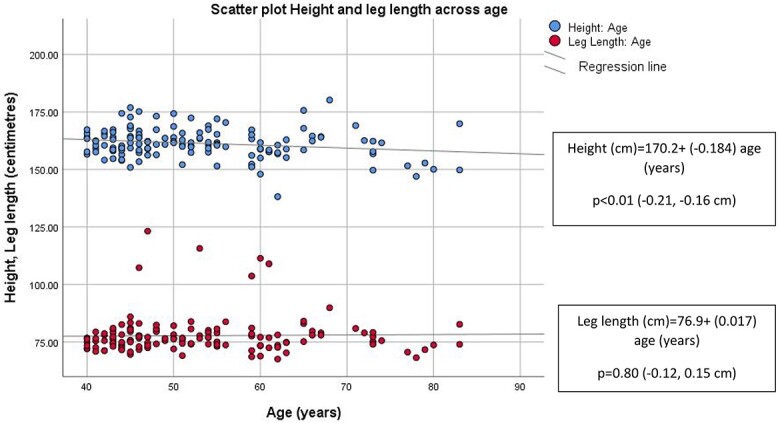
Leg length and height plotted across age (Cohort 1). Regression equations reported in corresponding boxes. *P* value (95% CI) relates to the significance of the relationship between variables examined in the regression analysis.

**Figure 2:**
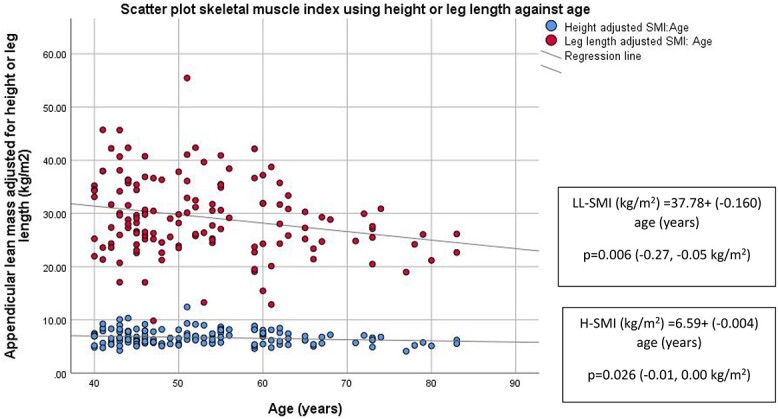
Scatter plot showing skeletal muscle mass index using height or leg length across age (Cohort 1). Regression equations are reported in corresponding boxes. *P* value (95% CI) relates to the significance of the relationship between variables examined in the regression analysis. LL-SMI: Leg length adjusted SMI, H-SMI: Height adjusted SMI.

Given the lack of difference observed in leg length between groups, a scatter plot was examined. Older women were shorter, while leg length remained unchanged across age (Figure 1). H-SMI and LL-SMI were significantly associated with age (Figure 2). Low LL-SMIs in older women were more apparent when visualised in the scatter plot.

##### Relationship between anthropometric variables to estimated   skeletal muscle mass by DEXA (Cohort 1)

Linear regression analysis was performed by examining the relationship between anthropometric measures to DEXA SMM. In a multiple regression analysis adjusted for age and mid-upper arm circumference, CC was the strongest predictor of DEXA SMM (*β*: 0.513, *r*^2^: 0.421, *P* < 0.001). For every 1 cm increase in CC, there was an increase in DEXA SMM by 0.51kg. 42.1% of variation to DEXA SMM was explained by age, mid-upper arm and CC (Supplementary Table 6).

To explore CC cut points corresponding to low skeletal muscle mass by EWGSOP2 definition, a ROC curve was created. When assessed against the EWGSOP2 criteria, a CC <35.60 cm provided a sensitivity of 88.6% and a specificity of 54.5% (AUC: 0.829, *P* < 0.001, 95% CI: 0.75–0.91) (Supplementary Figure 1).

When CC adjusted for leg length was explored, a CC adjusted for leg length index of 0.602 provided a sensitivity of 89.7% and a specificity of 61.8% when detecting low skeletal muscle mass by EWGSOP2 criteria (AUC: 0.736, *P* < 0.001, 95% CI: 0.64–0.84) (Supplementary Figure 2).

When CC adjusted for knee height was explored, an index of 0.135 provided a sensitivity of 85.7% and a specificity of 61.8% when detecting low skeletal muscle mass by EWGSOP2 criteria (AUC: 0.743, *P* < 0.001, 95% CI: 0.65–0.83) (Supplementary Figure 3).

### Cohort 2

This was a smaller cohort of 49 women age between 61 and 99 years. Data from this cohort were analysed to report on the relationship between H-SMI and LL-SMI to grip strength, a measure of muscle quality. DEXA data were available from 40 women. Mean age in this cohort was 76.7 ± 9.8 years old. Up to 36.7% of women in this cohort had low skeletal muscle mass by the EWGSOP2 criteria. Mean grip strength was 18.6 ± 7.6 kg.

**Figure 3:**
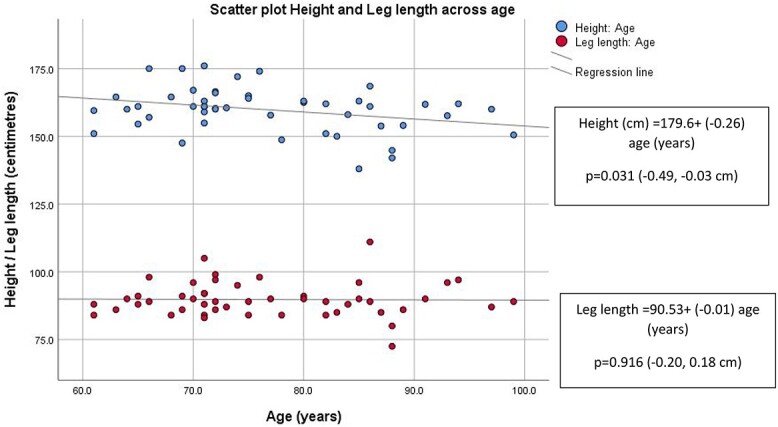
Scatter plot height and leg length across age (Cohort 2). Regression equations are reported in corresponding boxes. *P* value (95% CI) relates to the significance of the relationship between variables examined in the regression analysis.

**Figure 4:**
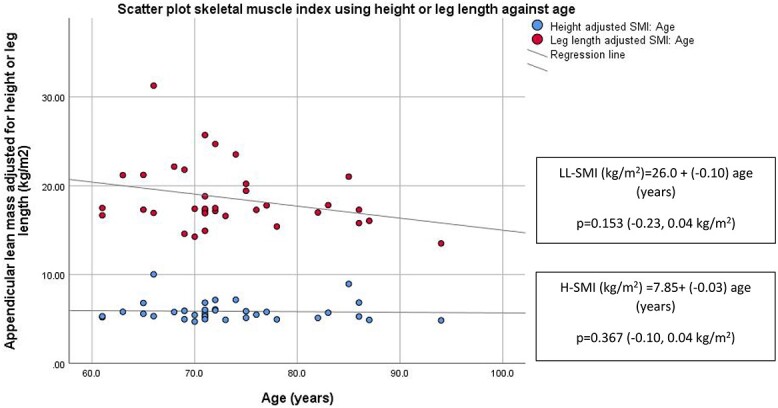
Scatter plot showing skeletal muscle mass index using height or leg length across age (Cohort 2). Regression equations are reported in corresponding boxes. *P* value (95% CI) relates to the significance of the relationship between variables examined in the regression analysis. LL-SMI: Leg length adjusted SMI, H-SMI: Height adjusted SMI.

A negative relationship was observed between age and height but not leg length. When both SMIs were examined in a scatter plot, older women had lower LL-SMI compared with relatively preserved H-SMI (Figures 3 and 4). Univariate linear regression analysis was performed to assess the relationship between H-SMI and LL-SMI to grip strength (Table 1). Both HH-SMI and LL-SMI had a positive relationship to grip strength, however when adjusted for age, only LL-SMI remained significant (*P* = 0.048).

## Discussion

Our study examined data from Australian women with the aim of evaluating an alternative SMI for skeletal muscle mass assessment. The relationship between anthropometric measures to skeletal muscle mass was also examined. We observed several findings.

### Older women have lower standing height while leg length was unchanged across age

Older women were demonstrated to have lower standing height while leg length was not different across age groups. Scatter plots comparing height and leg length show a significant negative relationship between height with age, contrasting with the lack of association between leg length and age in both cohorts. While a decline in height is expected, stable leg length measurements across age is a novel finding from our study. Further examination of scatter plots comparing both H-SMI and LL-SMI shows a more apparent decline in LL-SMI with age. In addition, following adjustment for age LL-SMI remained significantly associated with grip strength, supporting its role as a potential tool for skeletal muscle mass assessment.

**Table 1:** Linear regression analysis to grip strength as dependant variable (Cohort 2)

Independent variable	*r* ^2^	Beta	*P* value (95% CI)
H-SMI (kg/m^2^)	0.11	1.59	0.049 (0.01, 3.18)
LL-SMI (kg/m^2^)	0.15	0.71	0.014 (0.15, 1.26)
Age-adjusted model			
Independent variable	*r* ^2^	Beta	*P* value (95% CI)
H-SMI (kg/m^2^)	0.48	1.13	0.075 (−0.12, 2.39)
LL-SMI (kg/m^2^)	0.43	0.48	0.048 (0.004, 0.946)

Height was observed to decline with age in several longitudinal studies [9, 10, 23]. These changes can result in an artefactual increase in body mass index. Because of the decline in height proportional to skeletal muscle mass with increase age, there is also a propensity for H-SMI to underestimate low muscle mass particularly in the older population. Studies exploring the use of leg length in the SMI are scarce. Otsuka performed a longitudinal study incorporating several denominators in the SMI [24] in predicting mortality and disability. Compared to the conventional H-SMI, ALM adjusted for leg length was a better predictor of disability in men, while unadjusted ALM was a stronger predictor in women.

To our knowledge, we were unaware of studies in the Australian population examining the use of leg length in the SMI. Hence, our study finding provides new knowledge on the use of LL-SMI in a group of Australian women. While our analysis shows a significant relationship between LL-SMI and muscle strength, findings are limited to a small sample size. This novel finding would benefit from further exploration and validation of specific cut points for alternative SMI in a larger population. Additionally, assessing its relationship to outcome measures of muscle strength, disability and mortality would be pertinent in defining its role further.

### C‌C as a marker of low skeletal muscle mass

Anthropometrics are simple and reliable measurements, which can be performed at the bedside. Mid-upper arm [1] and CC [13, 25–28] have been explored as screening tools for low muscle mass. There is good correlation between CC and calf muscle by MRI [15], DEXA [13, 25, 29] and grip strength [30].

Increased CC was associated with low frailty index and reduced disability [31]. A CC of <31 cm was associated with impaired physical function, disability and lower frailty scores [14, 31]. Analysis from our study indicates that CC is a strong predictor of SMM, which is consistent with other study findings [15, 29].

When determining CC cut points in detecting low muscle mass by EWGSOP2 DEXA criteria, a CC < 35.60 cm provided a sensitivity of 88.6% and a specificity of 54.5%. This suggests this cut point is useful in detecting low skeletal muscle mass but cannot reliably exclude individuals without low muscle mass.

Given the ease of performing these measurements, we would still argue for its utility as a screening tool for sarcopenia detection in populations with impaired mobility (hospital patients and nursing home residents) or where access to skeletal muscle imaging is limited. To further expand work in this area, exploring the different cut points and correlations with outcome measures, such as disability and mortality, would provide further insights.

### SMI incorporating leg length and knee height as potential alternatives for skeletal muscle assessments

Obtaining accurate assessments of standing height in those with mobility impairment can be difficult and lead to imprecise results. These challenges have led us to consider whether there were other alternative measurements for the assessment of skeletal muscle mass. Knee height does not decline with age [32] and has been used to validate and predict standing height in the older population [33, 34]. As the majority of lean mass in the body is attributed to lean mass from the arms and legs, the use of leg lean mass adjusted for height, leg length and knee height was explored in our analysis.

In the comparison of alternative SMI between younger and older women using these measurements, there was a significant difference observed when lean mass was adjusted for leg length and knee height. These findings suggest leg length and knee height could be used as potential alternatives in those where accurate measurements of standing height may not be possible. An avenue for future research is the assessment of the relationship between these alternative skeletal muscle indices to muscle strength, function and clinical outcomes.

### Strengths and limitations

New findings from our study are that of the stable leg length across age groups and LL-SMI as a better indicator of skeletal muscle mass compared to the conventional H-SMI. In a small subset of women, LL-SMI was associated with grip strength, supporting its role as an alternative for skeletal muscle mass assessment.

Our study has several limitations. Data were obtained from a group of women with a variety of medical conditions limiting the generalizability of study findings to community-dwelling older women. There was also a lack of data on ethnicity and other confounding factors (nutritional status and lifestyle factors), which was not adjusted for in the analysis. In addition, body composition assessment in both cohorts differs by the use of different machines, and anthropometry data were only available from a select population in the cohort.

While we were able to show a significant relationship between LL-SMI and muscle strength this was performed on a small cohort. The lack of grip strength in the larger cohort limits further assessment between LL-SMI and measures of muscle quality, which would provide more meaningful results. Finally, there was also a difference in leg length measurement methods between both cohorts. However, it was considered that women from the hip fracture group were unlikely to provide accurate measures of sitting height, resulting in the difference in methods.

## Conclusion

To summarise, leg length was observed to remain stable across age. LL-SMI were shown to have a more obvious decline with age and were associated with grip strength. The use of LL-SMI may be a better alternative compared to H-SMI in skeletal muscle assessment in the older population. Alternative SMI using leg length and knee height can be useful alternative measures in populations where mobility is impaired and would benefit from further exploration in a multi-centre study to further delineate cut points and its relationship to meaningful outcomes and mortality.

## Supplementary Material

aa-21-1285-File002_afac002

## References

[1] Baumgartner RN, Koehler KM, Gallagher D et al. Epidemiology of sarcopenia among the elderly in New Mexico. Am J Epidemiol 1998; 147: 755–63. 9554417 10.1093/oxfordjournals.aje.a009520

[2] Rosenberg IH . Sarcopenia: origins and clinical relevance. J Nutr 1997; 127: 990S–1. 9164280 10.1093/jn/127.5.990S

[3] Cruz-Jentoft AJ, Bahat G, Bauer J et al. Sarcopenia: revised European consensus on definition and diagnosis. Age Ageing 2019; 48: 16–31. 30312372 10.1093/ageing/afy169 PMC6322506

[4] Studenski SA, Peters KW, Alley DE et al. The FNIH sarcopenia project: rationale, study description, conference recommendations, and final estimates. J Gerontol A Biol Sci Med Sci 2014; 69: 547–58. 24737557 10.1093/gerona/glu010 PMC3991146

[5] Chen LK, Liu LK, Woo J et al. Sarcopenia in Asia: consensus report of the Asian Working Group for Sarcopenia. J Am Med Dir Assoc 2014; 15: 95–101. 24461239 10.1016/j.jamda.2013.11.025

[6] Fielding RA, Vellas B, Evans WJ et al. Sarcopenia: an undiagnosed condition in older adults. Current consensus definition: prevalence, etiology, and consequences. International working group on sarcopenia. J Am Med Dir Assoc 2011; 12: 249–56. 21527165 10.1016/j.jamda.2011.01.003 PMC3377163

[7] Cruz-Jentoft AJ, Baeyens JP, Bauer JM et al. Sarcopenia: European consensus on definition and diagnosis: report of the European Working Group on sarcopenia in older people. Age Ageing 2010; 39: 412–23. 20392703 10.1093/ageing/afq034 PMC2886201

[8] Bhasin S, Travison TG, Manini TM et al. Sarcopenia definition: the position statements of the sarcopenia definition and outcomes consortium. J Am Geriatr Soc 2020; 68: 1410–8. 32150289 10.1111/jgs.16372 PMC12132920

[9] Cline MG, Meredith KE, Boyer JT, Burrows B. Decline of height with age in adults in a general population sample: estimating maximum height and distinguishing birth cohort effects from actual loss of stature with aging. Hum Biol 1989; 61: 415–25. 2807265

[10] Sorkin JD, Muller DC, Andres R. Longitudinal change in height of men and women: implications for interpretation of the body mass index: the Baltimore Longitudinal Study of Aging. Am J Epidemiol 1999; 150: 969–77. 10547143 10.1093/oxfordjournals.aje.a010106

[11] Kim YS, Lee Y, Chung YS et al. Prevalence of sarcopenia and sarcopenic obesity in the Korean population based on the Fourth Korean National Health and Nutritional Examination Surveys. J Gerontol A Biol Sci Med Sci 2012; 67: 1107–13. 22431554 10.1093/gerona/gls071

[12] Wen X, Wang M, Jiang CM, Zhang YM. Are current definitions of sarcopenia applicable for older Chinese adults? J Nutr Health Aging 2011; 15: 847–51.2215977110.1007/s12603-011-0088-3

[13] Kawakami R, Murakami H, Sanada K et al. Calf circumference as a surrogate marker of muscle mass for diagnosing sarcopenia in Japanese men and women. Geriatr Gerontol Int 2015; 15: 969–76. 25243821 10.1111/ggi.12377

[14] Rolland Y, Lauwers-Cances V, Cournot M. Sarcopenia, calf circumference, and physical function of elderly women: a cross-sectional study. J Am Geriatr Soc 2003; 51: 1120–4. 12890076 10.1046/j.1532-5415.2003.51362.x

[15] Asai C, Akao K, Adachi T et al. Maximal calf circumference reflects calf muscle mass measured using magnetic resonance imaging. Arch Gerontol Geriatr 2019; 83: 175–8. 31071533 10.1016/j.archger.2019.04.012

[16] Abreo AP, Bailey SR, Abreo K. Associations between calf, thigh, and arm circumference and cardiovascular and all-cause mortality in NHANES 1999-2004. Nutr Metab Cardiovasc Dis 2021; 31: 1410–5. 33762151 10.1016/j.numecd.2021.01.011

[17] Weng CH, Tien CP, Li CI et al. Mid-upper arm circumference, calf circumference and mortality in Chinese long-term care facility residents: a prospective cohort study. BMJ Open 2018; 8: e020485. 10.1136/bmjopen-2017-020485 PMC5942455 29743327

[18] Sousa IM, Bielemann RM, Gonzalez MC et al. Low calf circumference is an independent predictor of mortality in cancer patients: a prospective cohort study. Nutrition 2020; 79-80: 110816. 32569952 10.1016/j.nut.2020.110816

[19] Dent E, Chapman I, Piantadosi C, Visvanathan R. Screening for malnutrition in hospitalised older people: comparison of the mini nutritional assessment with its short-form versions. Australas J Ageing 2017; 36: E8–13. 10.1111/ajag.12402 28345773

[20] Dent E, Chapman I, Piantadosi C, Visvanathan R. Nutritional screening tools and anthropometric measures associate with hospital discharge outcomes in older people. Australas J Ageing 2015; 34: E1–6. 10.1111/ajag.12130 24444126

[21] Kim J, Heshka S, Gallagher D et al. Intermuscular adipose tissue-free skeletal muscle mass: estimation by dual-energy X-ray absorptiometry in adults. J Appl Physiol (1985) 2004; 97: 655–60. 15090482 10.1152/japplphysiol.00260.2004

[22] Lohman T, Roche A, Martorell R. In: Lohman T, Roche A, Martorell R, eds. Anthropometric Standardization Reference Manual. USA: Human Kinetics Books, 1998.

[23] Dey DK, Rothenberg E, Sundh V, Bosaeus I, Steen B. Height and body weight in the elderly. I. a 25-year longitudinal study of a population aged 70 to 95 years. Eur J Clin Nutr 1999; 53: 905–14. 10602346 10.1038/sj.ejcn.1600852

[24] Otsuka R, Matsui Y, Tange C et al. What is the best adjustment of appendicular lean mass for predicting mortality or disability among Japanese community dwellers? BMC Geriatr 2018; 18: 8. 29304751 10.1186/s12877-017-0699-6 PMC5756439

[25] Santos LP, Gonzalez MC, Orlandi SP et al. New prediction equations to estimate appendicular skeletal muscle mass using calf circumference: results from NHANES 1999-2006. JPEN J Parenter Enteral Nutr 2019; 43: 998–1007. 31081126 10.1002/jpen.1605

[26] Barbosa-Silva TG, Menezes AM, Bielemann RM, Malmstrom TK, Gonzalez MC, Grupo de Estudos em Composição Corporal e Nutrição (COCONUT). Enhancing SARC-F: improving sarcopenia screening in the clinical practice. J Am Med Dir Assoc 2016; 17: 1136–41. 27650212 10.1016/j.jamda.2016.08.004

[27] Mohd Nawi SN, Khow KS, Lim WS, Yu SC. Screening tools for sarcopenia in community-dwellers: a scoping review. Ann Acad Med Singapore 2019; 48: 201–16. 31495866

[28] Heymsfield SB, Martin-Nguyen A, Fong TM, Gallagher D, Pietrobelli A. Body circumferences: clinical implications emerging from a new geometric model. Nutr Metab (Lond) 2008; 5: 24. 18834550 10.1186/1743-7075-5-24 PMC2569934

[29] Gonzalez MC, Mehrnezhad A, Razaviarab N, Barbosa-Silva TG, Heymsfield SB. Calf circumference: cutoff values from the NHANES 1999-2006. Am J Clin Nutr 2021; 113: 1679–87. 33742191 10.1093/ajcn/nqab029 PMC8433492

[30] Yasuda T . Simplified morphological evaluation of skeletal muscle mass and maximum muscle strength in healthy young women: comparison between thigh and calf. Womens Health (Lond) 2020; 16: 1745506520962009. 33063630 10.1177/1745506520962009 PMC7580187

[31] Landi F, Onder G, Russo A et al. Calf circumference, frailty and physical performance among older adults living in the community. Clin Nutr 2014; 33: 539–44. 23948128 10.1016/j.clnu.2013.07.013

[32] Hurley RS, Bartlett BJ, Witt DD, Thomas A, Taylor EZ. Comparative evaluation of body composition in medically stable elderly. J Am Diet Assoc 1997; 97: 1105–9. 9336556 10.1016/S0002-8223(97)00270-8

[33] Pini R, Tonon E, Cavallini MC et al. Accuracy of equations for predicting stature from knee height, and assessment of statural loss in an older Italian population. J Gerontol A Biol Sci Med Sci 2001; 56: B3–7. 11193222 10.1093/gerona/56.1.b3

[34] Garcia-Pena C, Perez-Zepeda MU. Validity of knee-estimated height to assess standing height in older adults: a secondary longitudinal analysis of the Mexican health and aging study. J Nutr Health Aging 2017; 21: 262–5. 28244564 10.1007/s12603-016-0761-7 PMC5749405

