# Supplementary material for: Evaluation of an alternative skeletal muscle index for skeletal muscle mass assessment in a group of Australian women
Source: Age Ageing. 2022 Feb 12;51(2):afac002. doi: 10.1093/ageing/afac002 (PMC9160878; doi:10.1093/ageing/afac002)

**Supplementary Data: Evaluation of an alternative skeletal muscle index for skeletal muscle mass assessment in a group of Australian women.**

**SUPPLEMENTARY DATA**

Contents list:

1. Supplementary Table 1: Alternative skeletal muscle indices.
2. Supplementary Table 2: Cohort 1 demographics
3. Supplementary Table 3: Medical conditions for referral (Cohort 1)
4. Supplementary Table 4: Anthropometry data comparison (Cohort 1)
5. Supplementary Table 5: Comparison body composition data (Cohort 1)
6. Supplementary Table 6: Multiple regression analysis between anthropometric variables to skeletal muscle mass on DEXA (kg)
7. Supplementary Figure 1: ROC curve assessing cut points for calf circumference using EWGSOP2 criteria for low muscle mass.
8. Supplementary Figure 2: ROC curve assessing cut points for calf circumference adjusted for leg length using EWGSOP2 criteria for low muscle mass.
9. Supplementary Figure 3: ROC curve assessing cut points for calf circumference adjusted for knee height using EWGSOP2 criteria for low muscle mass.

Supplementary Table 1: Alternative skeletal muscle indices.

| Alternative skeletal muscle index | Height adjusted | Leg length adjusted | Knee height adjusted |
| --- | --- | --- | --- |
| Leg appendicular lean (ALM) mass | Leg lean mass (kg)/  Height (m^2^) | Leg lean mass (kg)/  Leg length (m^2^) | Leg lean mass (kg)/ Knee height (m^2^) |
| Calf circumference | Calf circumference (cm)/Height (m^2^) | Calf circumference (cm)/Leg length (m^2^) | Calf circumference (cm)/Knee height (m^2^) |

Supplementary Table 2: Cohort 1 demographics

| **Category** | **Whole Cohort**  n=2041 | **Group 1**  Age 40-60  n=1163 (56.9%) | **Group 2**  Age 61-92  n=878 (43.0%) | **p value** |
| --- | --- | --- | --- | --- |
| Age (years) (IQR) | 58 (51,68) | 52 (47,56) | 70 (65,76) | <0.001 |
| Standing Height (cm) ± SD | 159.4 ± 7.2 | 160.9 ± 6.9 | 157.3± 7.1 | <0.001 |
| Weight (kg) (IQR) | 65.8 (58.0, 74.7) | 65.4 (57.9, 75.3) | 66.1 (58.0, 74.1) | 0.496 |
| Body mass index (kg/m^2^) (IQR) | 25.9 (22.9, 29.4) | 25.3 (22.6, 28.9) | 26.9 (23.5, 29.8) | <0.001 |

Supplementary Table 3: Medical conditions for referral (Cohort 1)

| **Category** | **Frequency** | **Percent (%)** |
| --- | --- | --- |
| Screening for osteoporosis | 509 | 24.9 |
| Previous fracture / Established Osteoporosis | 341 | 16.7 |
| Premature menopause | 327 | 16.0 |
| Breast cancer | 229 | 11.2 |
| Steroid use | 168 | 8.2 |
| Others (Hypogonadal, anticonvulsant use) | 131 | 6.4 |
| Rheumatological condition | 94 | 4.6 |
| Thyroid dysfunction, hyperparathyroidism | 83 | 4.1 |
| Chronic Kidney Disease | 43 | 2.1 |
| Post-transplant | 38 | 1.9 |
| Coeliac Disease | 31 | 1.5 |
| Chronic liver disease | 20 | 1.0 |
| Diabetes | 11 | 0.5 |
| Turners Syndrome | 11 | 0.5 |
| Bariatric Surgery | 5 | 0.2 |

Supplementary Table 4: Anthropometry data comparison (Cohort 1)

| **Anthropometry Data Comparison** | | | | |
| --- | --- | --- | --- | --- |
| **Category** | **Whole cohort** | **Group 1**  Age 40-60 | **Group 2**  Age 61-100 | **p value** |
| Standing Height (cm) ± SD | 159.4 ± 7.2 | 160.9 ± 6.9 | 157.3± 7.1 | <0.001 |
| Sitting height (cm) (IQR) | n=137  85.4  (82.6, 87.5) | n=106  85.9  (83.1,88.4) | n=31  83.6  (80.9, 85.7) | 0.004 |
| Leg length (cm) (IQR) | n=137  76.5  (73.7, 79.0) | n=106  76.6  (73.7,79.0) | n=31  75.6  (73.7,79.1) | 0.837 |
| Waist circumference (cm) (IQR) | n=124  83.0  (76.0,96.5) | n=86  83.0  (76.0,97.0) | n=38  83.5  (77.0,94.0) | 0.569 |
| Mid upper arm circumference (cm) (IQR) | n=123  28.0  (25.4,32.0) | n=86  28.6  (25.7,32.7) | n=37  27.0  (24.1,30.5) | 0.030 |
| Gluteal circumference (cm) (IQR) | n=124  97.5  (91.0,105.5) | n=86  99.0  (92.0,106.0) | n=38  95.0  (90.0,105.0) | 0.466 |
| Thigh circumference (cm) (IQR) | n=112  48.4  (44.5,53.9) | n=79  51  (45.9,55.6) | n=33  45.0  (42.6,51.0) | 0.001 |
| Calf circumference (cm) (IQR) | n=112  34.6  (32.5, 37.9) | n=80  35.4 (33.5,38.4) | n=32  33.0  (31.4, 35.10) | 0.002 |

Supplementary Table 5: Comparison body composition data (Cohort 1)

| **Body composition and bone mineral density data by DEXA** | | | | |
| --- | --- | --- | --- | --- |
| **Category** | **Whole cohort**  (n=2041) | **Group 1**  Age 40-60  n=1163 | **Group 2**  Age 61-100  n=878 | **p value** |
| **Body composition** | | | | |
| DEXA ALM (kg) (IQR) | 15.50  (14.00,17.10) | 15.81  (14.22, 17.37) | 15.10  (13.63, 16.66) | <0.001 |
| Estimated DEXA SMM (kg) (IQR) | 17.30  (15.50,19.20) | 17.80  (16.00, 19.55) | 16.70  (15.00, 18.50) | <0.001 |
| DEXA fat mass (kg) (IQR) | 25.57  (19.60, 32.51) | 24.76  (19.26,32.32) | 26.41  (20.31, 32.77) | 0.151 |
| DEXA fat mass index (kg/m^2^) (IQR) | 10.11  (7.78,12.87) | 9.70  (7.43, 12.50) | 10.74  (8.20, 13.14) | <0.001 |
| **Bone mineral density** | | | | |
| DEXA BMC (kg) (IQR) | 2.26  (2.00, 2.56) | 2.37  (2.10, 2.65) | 2.13  (1.86, 2.39) | <0.001 |
| DEXA total body BMD (gm/cm^2^) (IQR) | 1.09  (1.02,1.16) | 1.12  (1.06,1.18) | 1.04  (0.97,1.11) | <0.001 |
| DEXA femoral neck BMD (gm/cm^2^) (IQR) | 0.85  (0.76, 0.95) | 0.90  (0.82, 0.99) | 0.78  (0.70, 0.88) | <0.001 |
| DEXA femoral neck T score (IQR) | -1.05  (-1.80, -0.20) | -0.60  (-1.30, 1.00) | -1.60  (-2.20, -0.80) | <0.001 |
| **Skeletal muscle index (height adjusted)** | | | | |
| Appendicular lean (kg) adjusted for height (m^2^) (H-SMI) (IQR) | n=2041  6.10  (5.65, 6.59) | n=1163  6.09  (5.64, 6.58) | n=878  6.13  (5.66, 6.61) | 0.528 |
| **Alternative skeletal muscle index** | | | | |
| Appendicular lean adjusted for leg length (LL-SMI) (kg/m^2^) ± SD | n=137  29.30 ± 0.62 | n=106  30.16±7.50 | n=31  26.38±5.20 | 0.002 |
| Appendicular lean adjusted for knee height (kg/m^2^) (IQR) | n=111  49.3  (47.3, 51.0) | n=77  49.3  (47.6, 50.9) | n=34  49.1  (46.8, 51.5) | 0.013 |
| Leg ALM adjusted for height (kg/m^2^)  ± SD | n=111  4.45 ± 0.59 | n=77  4.47 ± 0.58 | n=34  4.40 ± 0.62 | 0.549 |
| Leg ALM adjusted for leg length (kg/m^2^) ± SD | n=137  21.93 ± 5.43 | n=106  22.56 ± 5.66 | n=31  19.76 ± 3.90 | 0.002 |
| Leg ALM adjusted for knee height (kg/m^2^) ± SD | n=111  47.6 ± 6.53 | n=77  48.6 ± 6.10 | n=34  42.9 ± 7.02 | 0.018 |
| Calf circumference (m) adjusted for height (m^2^) ± SD | n=112  0.14 ± 0.02 | n=80  0.14 ± 0.02 | n=32  0.14 ± 0.14 | 0.982 |
| Calf circumference (m) adjusted for leg length (m^2^) (IQR) | n=94  0.60  (0.53, 0.67) | n=68  0.60  (0.54, 0.69) | n=26  0.57  (0.53, 0.62) | 0.199 |
| Calf circumference (m) adjusted for knee height (m^2^) ± SD | n=105  1.45 ± 0.18 | n=76  1.47 ± 0.17 | n=29  1.39 ± 0.20 | 0.042 |
| **Low skeletal muscle mass by DEXA criteria** | | | | |
| EWGSOP 2, n (%) | n=420  (20.6%) | n=239  (20.6%) | n=181  (20.6%) | 0.971 |

DEXA SMM: DEXA skeletal muscle mass, DEXA ALM: DEXA Appendicular Lean Mass, DEXA SMI: DEXA Skeletal muscle mass index, DEXA BMC: DEXA Bone Mineral Content, DEXA total body BMD: DEXA total body bone mineral density, DEXA femoral neck BMD: DEXA femoral neck bone mineral density, Leg ALM: Leg appendicular lean mass, EWGSOP 2: European Working Group on Sarcopenia in Older People 2

Supplementary Table 6: Multiple regression analysis between anthropometric variables to skeletal muscle mass on DEXA (kg)

| **Independent variable** | **r^2^** | **Beta** | **p value (95% CI)** |
| --- | --- | --- | --- |
| Age | 0.421 | -0.022 | 0.237 (-0.060,0.015) |
| Mid upper arm circumference |  | 0.002 | 0.969 (-0.114,0.118) |
| Calf circumference |  | 0.513 | <0.001 (0.362,0.664) |

Supplementary Figure 1: ROC curve assessing cut points for calf circumference using EWGSOP2 criteria for low muscle mass.


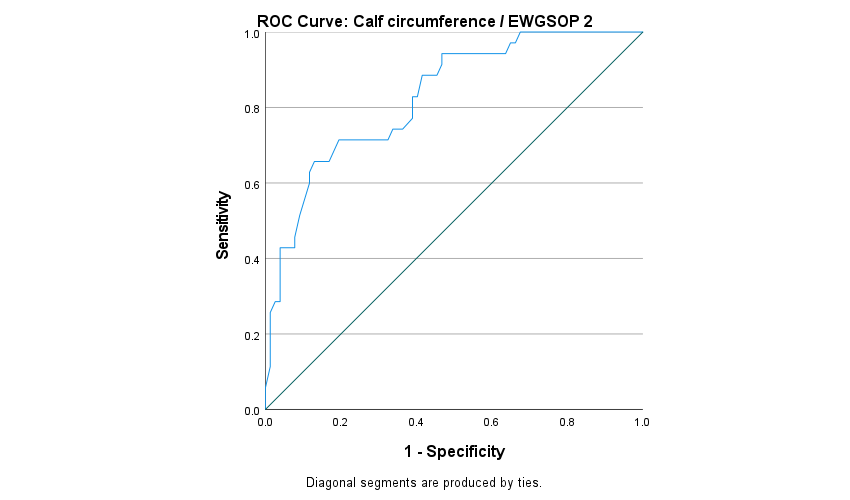


Supplementary Figure 2: ROC curve assessing cut points for calf circumference adjusted for leg length using EWGSOP2 criteria for low muscle mass.


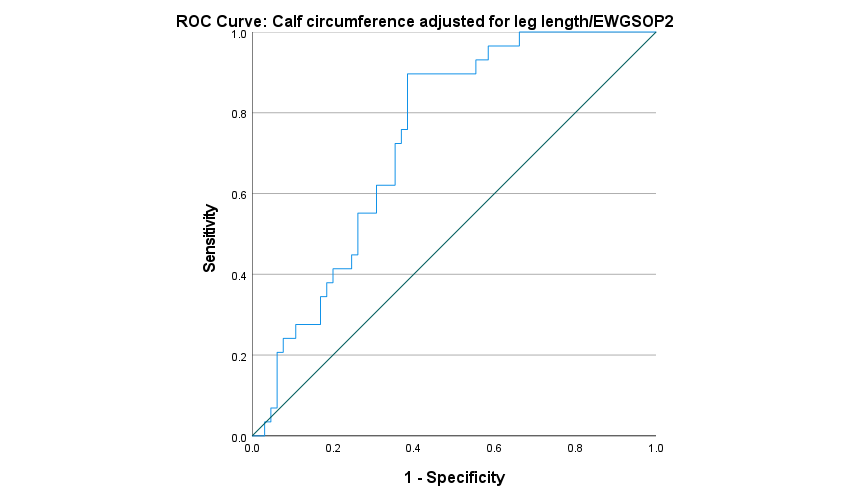


Supplementary Figure 3: ROC curve assessing cut points for calf circumference adjusted for knee height using EWGSOP2 criteria for low muscle mass.


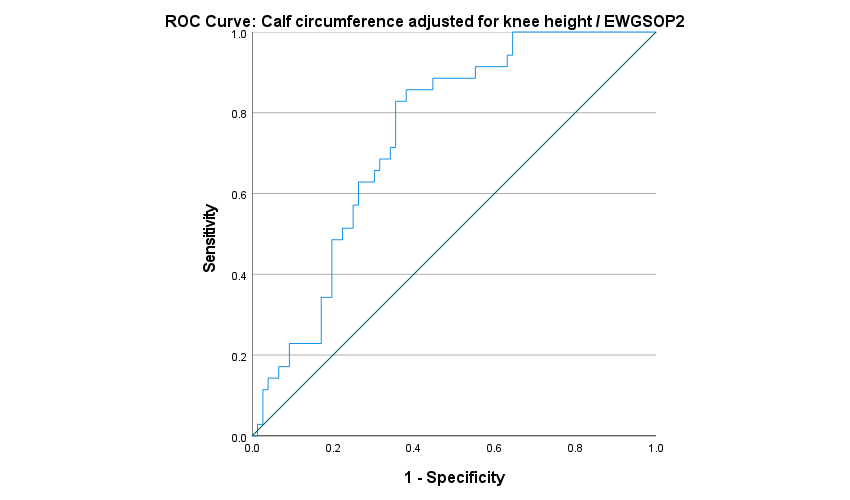

Supplement: aa-21-1285-File002_afac002 [file aa-21-1285-file002_afac002.docx]
